# On the move, direction matters: Polar localization of OsLsi1 for differential uptake of metalloids in rice

**DOI:** 10.1093/plphys/kiaf262

**Published:** 2025-06-19

**Authors:** Munkhtsetseg Tsednee

**Affiliations:** Assistant Features Editor, Plant Physiology, American Society of Plant Biologists; Research Center for Environmental Changes, Academia Sinica, Taipei 11529, Taiwan

Plants acquire mineral nutrients from soil via plasma membrane–localized transporters. Thereafter, transport occurs across sequential layers of cells, including the epidermis, exodermis, and endodermis, and engages both influx and efflux pathways (Huang et al. 2024). However, some transporters, such as the silicon (Si) transporter in rice (OsLsi1) and boron (B) transporters in Arabidopsis (AtNIP5;1 and AtBOR1), display polar localizations at either the distal or the proximal side of the specific cell layers (Yoshinari et al. 2016; Wang et al. 2017; Konishi et al. 2023). Although these gene products are well-known to facilitate metalloid transport, the impact of their polar localizations on the transport of different metalloids is unclear.

In this issue of *Plant Physiology*, Konishi et al. (2025) investigated the role of the polar localization of OsLsi1 transporter for multiple metalloid uptake and revealed its critical requirement in a coordinated and directional transport of metalloids. It is common for metalloid transporters to pass several different elements because of their similar chemical properties. For example, OsLsi1 transports germanic acid (Ge), arsenite (As), selenite (Se), antimonite (Sb), and boric acid in addition to silicic acid in the form of noncharged molecules at pH below 9.0 (Ma and Yamaji 2015). The iron transporter AtIRT1 also transports zinc, cadmium, manganese, and cobalt besides iron (Barberon et al. 2014).

Konishi et al. (2025) made use of rice lines that they previously generated with OsLsi1 expressed in different polar distributions (Konishi et al. 2023). They first observed a full recovery of the reduced accumulation of metalloids, including B, As, Ge, Sb, and Se, in *lsi1-3* mutant shoots by the introduction of polar-localized OsLsi1. The nonpolar localized OsLsi1 resulted in varied accumulations of metalloids in shoots, with a decrease (for B, As, and Ge), an increase (for Sb), and no change (for Se). This observation suggested that the polar localization of OsLsi1 differentially affects the transport of different metalloids.

The authors focused on B and detected varying levels of B uptake in lines harboring nonpolar localized OsLsi1, depending on the B supply. As observed in the polarly localized OsLsi1 lines, rice maintains its internal B levels in response to fluctuations in external B. However, nonpolar localized OsLsi1 lines exhibited a loss of such internal B maintenance, showing reduced and increased B accumulations in shoots under low and high B, respectively. B transport activity was similar in both OsLsi1 variants (Konishi et al. 2025). Therefore, the authors proposed that the alternations in B uptake and accumulation in nonpolar localized OsLsi1 lines might have resulted from the contributions of the cooperating efflux transporter and their expression levels.

In rice roots, the OsBOR1 efflux transporter partners with OsLsi1 to mediate B transport toward central vasculature (Ma and Yamaji 2015). At the transcriptional level, neither *OsLsi1* nor *OsBOR1* genes respond to external B levels (Ma and Yamaji 2015). However, the OsBOR1 protein degrades in response to high B, while the OsLsi1 protein abundance remains unchanged under different B supplies (Ma and Yamaji 2015; Yoshinari et al. 2016).

In all OsLsi1 lines, the authors observed comparable mRNA accumulations for both *OsLsi1* and *OsBOR1* genes and similar protein abundance for OsLsi1 under low and high B. The OsBOR1 protein abundance decreased with increasing B supplies and was not detected in all lines under high B. As expected, the OsLsi1 showed polar distributions at the distal side of root exodermis and endodermis in polar localized OsLsi1 lines, but not in nonpolar localized OsLsi1 lines. The OsBOR1 was also located polarly at the proximal side of exodermis and endodermis in both OsLsi1 variants under low B. These results indicated that the loss of polar localization of OsLsi1 did not affect the OsBOR1 polar localization and its degradation under high B.

Since the B transport activity of OsLsi1 as well as the mRNA and protein abundances of 2 transporters remains similar between polar and nonpolar localized OsLsi1 lines, it is plausible that the presence or absence of OsBOR1 might itself have contributed to the altered B uptake and accumulation in nonpolar localized OsLsi1 variants. Moreover, OsLsi1 exhibits bidirectional transport activity for metalloids depending on their gradient concentrations (Mitani et al. 2008).

Polarly localized OsLsi1, at the distal side of the exodermis and endodermis, forms a directional transport pathway with efflux transporters (OsBOR1 for B and OsLsi2 for Si, Ge, and As) and facilitates the uptake of metalloids (Figure. A). However, when OsLsi1 loses its polar localization and locates also at the proximal side, it reimports the metalloids, transported by the efflux transporters to the apoplastic space, and thereby decreases the efficiency of the uptake (Figure. B). In the cases of Sb and high B, due to a lack of an efflux transporter or downregulation of OsBOR1, nonpolar localized OsLsi1 at the proximal side facilitates the flow of Sb and B toward the stele side, resulting in increased uptake of these metalloids (Figure. C).

**Figure.**
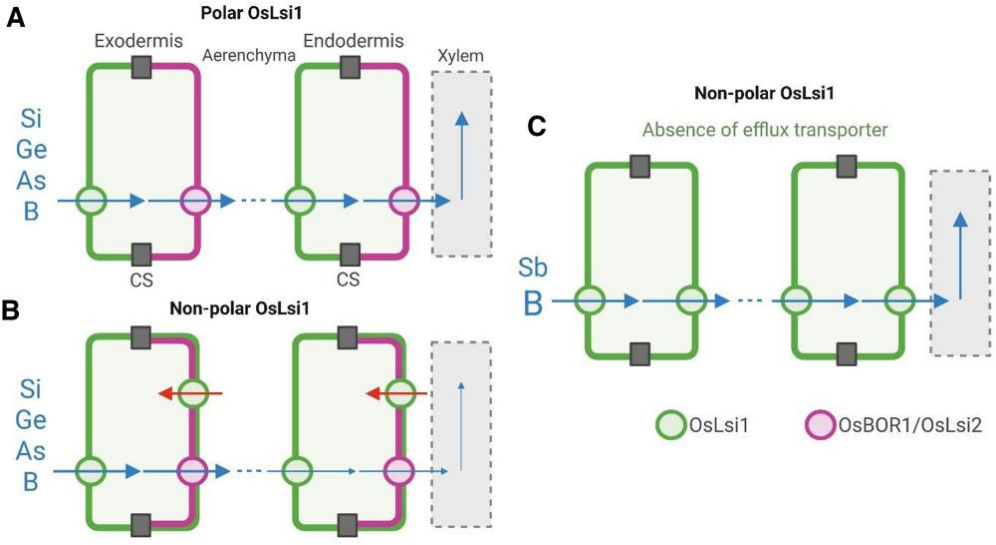
Schematic presentation on the role of OsLsi1 polar localization in metalloid uptake in rice. **A)** When OsLsi1 is polarly localized at the distal side of the exodermis and endodermis, it forms a directional transport pathway with efflux transporters, facilitating the uptake of metalloids, including Si, Ge, As, and B. **B)** However, when OsLsi1 loses its polar localization, these metalloids, transported by the efflux transporters to the apoplastic space, are re-taken up by OsLsi1 localized at the proximal side, decreasing the efficiency of the uptake. **C)** In the cases of an absence of efflux transporter (for Sb) or downregulation of OsBOR1 (at high B conditions), nonpolar OsLsi1 at the proximal side facilitates the flow of Sb and B toward the stele side, resulting in increased uptake of these metalloids. Green indicates OsLsi1 localization, and pink indicates localization of efflux transporters. Arrows indicate the flows of metalloids from the soil to the steel (blue) and the reuptake of metalloids (red). CS, Casparian strips. The figure was modified from Konishi et al. (2025).

In summary, Konishi et al. (2025) have revealed the critical role of the polar localization of OsLsi1 for the directional uptake of metalloids in rice, depending on the presence and absence of the cooperating efflux transporters. This finding highlights the importance of precise polar localization of the transporter for metalloid transport in plants.

## Data Availability

There are no data associated with this study.

## References

[kiaf262-B1] Barberon M, Dubeaux G, Kolb C, Isono E, Zelazny E, Vert G. Polarization of IRON-REGULATED TRANSPORTER 1 (IRT1) to the plant-soil interface plays crucial role in metal homeostasis. Proc Natl Acad Sci U S A. 2014:111(22):8293–8298. 10.1073/pnas.1402262111 24843126 PMC4050562

[kiaf262-B2] Huang S, Yamaji N, Ma JF. Metal transport systems in plants. Annu Rev Plant Biol. 2024:75(1):1–25. 10.1146/annurev-arplant-062923-021424 38382903

[kiaf262-B3] Konishi M, Mitani-Ueno M, Ma J. Role of polar localization of the silicon transporter OsLsi1 in metalloid uptake by rice roots. Plant Physiol. 2025:198(1);kiaf196. 10.1093/plphys/kiaf196 40341966 PMC12089981

[kiaf262-B4] Konishi N, Mitani-Ueno N, Yamaji N, Ma JF. Polar localization of a rice silicon transporter requires isoleucine at both C- and N-termini as well as positively charged residues. Plant Cell. 2023:35(6):2232–2250. 10.1093/plcell/koad073 36891818 PMC10226592

[kiaf262-B5] Ma JF, Yamaji N. A cooperative system of silicon transport in plants. Trends Plant Sci. 2015:20(7):435–442. 10.1016/j.tplants.2015.04.007 25983205

[kiaf262-B6] Mitani N, Yamaji N, Ma JF. Characterization of substrate specificity of a rice silicon transporter, Lsi1. Pflugers Arch. 2008:456(4):679–686. 10.1007/s00424-007-0408-y 18214526

[kiaf262-B7] Wang SL, Yoshinari A, Shimada T, Hara-Nishimura I, Mitani-Ueno N, Ma JF, Naito S, Takano J. Polar localization of the NIP5;1 boric acid channel is maintained by endocytosis and facilitates boron transport in arabidopsis roots. Plant Cell. 2017:29(4):824–842. 10.1105/tpc.16.00825 28341806 PMC5435427

[kiaf262-B8] Yoshinari A, Fujimoto M, Ueda T, Inada N, Naito S, Takano J. DRP1-Dependent endocytosis is essential for polar localization and boron-induced degradation of the borate transporter BOR1 in. Plant Cell Physiol. 2016:57(9):1985–2000. 10.1093/pcp/pcw121 27449211

